# Effects of Photoperiod on the Performance, Blood Profile, Welfare Parameters, and Carcass Characteristics in Broiler Chickens

**DOI:** 10.3390/ani12172290

**Published:** 2022-09-03

**Authors:** Hee-Jin Kim, Jiseon Son, Jin-Joo Jeon, Hyun-Soo Kim, Yeon-Seo Yun, Hwan-Ku Kang, Eui-Chul Hong, Ji-Hyuk Kim

**Affiliations:** 1 Poultry Research Institute, National Institute of Animal Science, RDA, Pyeongchang 25342, Korea; 2 Department of Animal Resources Science, Kongju National University, Yesan 32439, Korea

**Keywords:** photoperiod, growth, welfare, carcass, broiler

## Abstract

**Simple Summary:**

The physiology and behavior of birds, including poultry, are greatly affected by light. The poultry industry uses lighting regimens with long lighting periods to maximize the growth of broiler chickens. Rapid growth not only adversely affects the health of the birds, but also causes great stress. As animal welfare issues have become increasingly important, concerns regarding the current photoperiods in broiler production have increased. In this study, the effects of the photoperiod on the productivity and various welfare indicators on broiler rearing were investigated. Generally, the productivity and stress of broilers were both positively and negatively correlated with the photoperiod. The results showed that a photoperiod of 18L:6D was appropriate, when considering the growth and stress of broilers. The optimum photoperiod for broilers may vary in different feeding phases, with respect to performance and welfare. Therefore, continuous research is needed to establish the ideal light regimens under the consideration of the productivity and welfare of broilers.

**Abstract:**

We studied the effects of photoperiods on the growth performance, blood profile, welfare parameters, and carcass characteristics of broilers. A total of 336 male broiler chicks (Ross 308) were randomly allocated into 4 treatments (84 birds per treatment with 4 replicates), based on the following lighting regimen: 24 h continuous light (24L), 18 h continuous light (18L:6D), 8 h continuous light (8L:16D), and intermittent light (4L:2D). Body weight and feed intake of 7- and 35-day-old broilers were measured. At 5 weeks of age, 12 birds per treatment were selected for blood collection and carcass analysis. Body weight, body weight gain, and feed intake were the lowest in the 8L:16D treatment (*p* < 0.05). The heterophil-to-lymphocyte ratio, aspartate aminotransferase, interleukin-6, and corticosterone levels in the 24L treatment increased significantly when compared to that in the 18L:6D treatment (*p* < 0.05). The footpad dermatitis score was significantly lower in the 18L:6D and 8L:16D treatments than in the 24L and 4L:2D treatments (*p* < 0.001). There was no significant difference in the carcass and meat characteristics, except for the shear force of breast meat (*Pectoralis major*), which was the lowest in the 8L:16D treatment (*p* < 0.05). These results indicate that a photoperiod of 18 h resulted in an improvement in the performance and welfare of birds and a simultaneous decrease in stress level. However, further research is needed to establish a lighting regimen that satisfies both the productivity and welfare requirements of broilers in different feeding phases.

## 1. Introduction

Light is a critical extrinsic factor that controls physiological and behavioral processes in birds, especially poultry, where birds are often grown in artificial light environments. The characteristics of light, such as intensity, spectrum (or wavelength), and photoperiod affect living organisms [1], and are used as management tools to control behavior and improve the well-being and performance of poultry [2]. Photoperiod, along with light intensity, is an important factor that influences the physical growth and reproductive development of birds. The photoperiodic regimens of broiler chickens can be manipulated to maximize feed intake and efficiency [3].

Typical photoperiods include continuous light (24L), near-continuous light (23L:1D), intermittent light with repeating light and dark cycles, and step-up light with a gradually increasing photoperiod [1]. Since rapid growth and high feed efficiency are important parameters for the productivity and economy of the broiler industry, most broiler facilities use the continuous or intermittent light regimens that help maximize the feed intake and growth of broilers [4,5,6]. Although conventional lighting regimens induce rapid growth and improve the performance of broilers, they can also cause several health effects and raise welfare concerns [7]. Rapid growth increases the incidence of skeletal disorders and metabolic diseases, which may increase the mortality of broilers [1].

Reducing the growth rate by controlling the photoperiod may reduce the incidence of skeletal and metabolic diseases. Classen and Riddell [3] reported that a gradual increase in photoperiod from 6 to 23 h significantly reduced leg abnormalities in broilers, compared with those in a control group that received continuous light for 23 h; both groups exhibited similar performance parameters. Several studies have suggested the use of a more natural light pattern instead of the commonly used 23L:1D regimen to limit skeletal abnormalities in broilers [6,8,9].

A moderate photoperiod (i.e., 16L:8D) is beneficial for the welfare of broilers, including reduced physiological stress, enhanced immune response, and increased sleep duration and physical activity [9,10,11].

Darkness is as important as light for the growth and health of birds [7]. Prolonged dark periods during rearing impede access to feed, which further leads to reduced feed intake and limited growth. However, dark periods provide sufficient resting time for the birds, allowing reduced stress levels that help their health [4,12,13]. Rest and sleep serve vital functions, such as tissue restoration and growth, energy conservation, neurobehavioral performance, etc. [14]. Animal welfare organizations, such as the Royal Society for the Prevention of Cruelty to Animals (RSPCA) [15] and Animal Welfare Certification Standards (AWCS) [16], recommend a minimum dark period of 6 h as the poultry welfare standard.

Stress parameters that can indicate animal welfare status include blood corticosterone, blood glucose, triglycerides, and the heterophils-to-lymphocytes (H/L) ratio. The incidence of footpad dermatitis (FPD) is also used as a welfare parameter in broilers reared in floor-houses [13]. Animal welfare is a major issue worldwide, and measures to improve the welfare of chickens are implemented in the poultry industry. Although several reports have been published on the influence of lighting regimens on the performance and health conditions of broilers, only few studies involve a comprehensive analysis of parameters such as productivity, various stress indicators, and carcass characteristics. Therefore, the purpose of the present study was to compare the effects of different photoperiods on productivity, blood biochemical profiles, stress and welfare indicators, and carcass characteristics of broilers.

## 2. Materials and Methods

### 2.1. Birds, Housing and Experimental Design

Ross 308 male broilers (336, 1-day-old, body weight 48.0 ± 0.17 g) were used in this study. The experiment was performed in a floor-pen broiler house at the Poultry Research Institute (Pyeongchang, Korea) for 35 days. For the first 7 days, the chicks were reared under the same light intensity (30 lx) and photoperiod (22L:2D). From the second week, the chicks were randomly allocated to four treatments (4 replicates per treatment, 21 birds per replicate). The four treatments were 24 h continuous light (24L), 18 h continuous light (18L:6D), 8 h continuous light (8L:16D), and intermittent light (4L:2D). The 18L:6D and 8L:16D treatments were set based on AWCS of Korea [16], which recommends a minimum dark period of 6 h and a maximum light period of 8 h.

The birds were provided with a corn-soybean meal-based commercial broiler starter diet (CP 22.5%, ME 3020 kcal/kg) for week 1, a grower diet (CP 18.5%, ME 3050 kcal/kg) for weeks 2–3, and a finisher diet (CP 18.0%, ME 3100 kcal/kg) for weeks 4–5. Throughout the experiment, the birds had ad libitum access to feed, and water via a bell-type water dispenser. An LED light bulb was used as the light source, and the light intensity was maintained at 35 lx.

### 2.2. Growth Performance

For growth performance analysis according to photoperiod, body weight was measured on the initial (7-days-old) and final days (35-days-old) to determine the body weight gain and feed conversion ratio. The feed intake for each treatment was calculated by measuring the remaining feed at the end of the experiment. The feed conversion ratio was defined as the ratio of feed intake to weight gain during the test period.

### 2.3. Blood Sampling and Measurements

At 35 days, blood samples were collected from the wing veins of 12 birds (3 birds per replication, birds of similar body weight) selected from each treatment. Blood samples were analyzed for leukocytes, erythrocytes, and thrombocytes using a hemocytometer (HematVet 950; Drew Scientific, FL, USA). The biochemical composition of the serum was analyzed using a hematology analyzer (AU480 Chemistry Analyzer, Beckman Coulter Inc., CA, USA).

### 2.4. Cytokines and Corticosterone

Corticosterone and cytokine stress hormone levels were analyzed in the serum samples. Cytokine analysis was performed using tumor necrosis factor-α (TNF-α) and interleukin-6 (IL-6) with the Chicken IL-6 ELISA Kit (MBS268769, MyBioSource, San Diego, CA, USA) and Chicken TNF-α ELISA Kit (MBS2509660, MyBioSource, San Diego, CA, USA), respectively. Corticosterone analysis was performed using a commercially available Chicken Corticosterone Kit (ECH0077, Wuhan Fine Biotech Co. Ltd., Wuhan, China), according to the manufacturer’s instructions.

### 2.5. Litter Moisture Content and Footpad Dermatitis

To determine the moisture content of litter, 40 g of litter was collected on the day before slaughter; dry weight measurements were collected after drying the litter samples for 24 h at 105 °C. Moisture content was expressed as a percentage (%) of the difference between the fresh weight and dry weight of litter samples.

The footpad dermatitis (FPD) score of the broilers was evaluated according to the criteria of Welfare Quality [17]. The FPD score ranged from 0 to 4 points (Table 1); 40 birds per treatment were evaluated during the final week. The FPD score was calculated as the average value of the degrees.

### 2.6. Carcass Yield and Carcass Cut Yields

At 35 days of age, 12 birds per treatment (3 birds per replicate) were selected for the measurement of processing performance. The selected birds were euthanized via CO_2_ asphyxiation and the carotid artery was subsequently cut and exsanguinated. The feathers, head, feet, and intestines were removed to measure the carcass yield. Carcass cut yields were measured by dividing the breasts, legs, wings, neck, and back. Carcass yield and carcass cut yields were expressed as a percentage of carcass weight and partial meat weight to the living body weight.

### 2.7. Physicochemical Properties of Breast Meat

Breast meat (*Pectoralis major*) samples collected from 12 birds per treatment were used to analyze the physicochemical parameters. The samples were weighed and stored at 4 °C until 24 h postmortem. The chemical composition (moisture, crude protein, crude fat, and crude ash) of the breast meat was determined using the AOAC method [18]. The pH was measured using a pH meter (pH-K21, NWK-Binar GmbH, Celiusstr, Germany) and color intensity (CIE L*, a*, b*) was measured using a colorimeter (CR301 Chromameter, Minolta Co., Japan), calibrated with a white standard plate (Y = 92.40, x = 0.3136, and y = 0.3196).

The shear force (SF), cooking loss and water holding capacity (WHC) of breast meat were analyzed according to method of Chae et al. [19]. For the determination of SF, each sample (average weight, 61 g) was heated individually in a polyethylene bag immersed in a water bath at 70 °C for 10 min. The samples were then cooled at room temperature, and cores (diameter, 1.27 cm) were collected in the longitudinal direction of the muscle fibers. SF values were estimated using a Warner–Bratzler shear blade attached to a texture analyzer (TA-XT2, Stable Micro Systems Ltd., Surrey, UK). To measure the cooking loss, each sample was placed in a polyethylene bag that was then heated at 85 °C in a water bath for 45 min. After cooling at room temperature for 20 min, cooking loss was calculated as the percentage of weight loss after heating. WHC was calculated as a percentage of the difference between them by subtracting the free water generated by centrifugation and the total water in the meat. For free water, 0.5 g of a sample from which fat and fascia (tendon) were removed was placed in a tube, heated at 80 °C for 20 min in a water bath, and centrifuged at 448× *g* for 10 min. WHC percentage was calculated as the value obtained by dividing the fat coefficient (the value obtained after subtracting the fat content from the sample, %, n = 12) by the weight before and after centrifugation.

### 2.8. Statistical Analysis

All data were analyzed using the general linear model (GLM) procedure of SAS software (version 9.4, SAS Institute, Cary, NC, USA). Duncan’s multiple range test was used to determine significant differences among treatments. Differences were considered statistically significant at *p* < 0.05.

## 3. Results and Discussion

### 3.1. Growth Performance 

Table 2 shows the body weight, feed intake and feed conversion ratio of broilers according to the lighting regimen. Final body weight and body weight gain were the lowest in the 8L:16D treatment at 1862 and 1705 g, respectively (*p* < 0.05). However, no significant difference was observed between the 8L:16D and 18L:6D treatments. Schwean-Lardner et al. [20] reported that feed intake decreases at photoperiods less than 18L:6D. In this study, feed intakes in the 18L:6D and 8L:16D treatments were 3315 and 3117 g, respectively, indicating a decreasing trend when the photoperiod was less than 18L:6D. However, the feed conversion ratio did not differ significantly among the treatments.

### 3.2. Blood Cell Composition

Table 3 shows the results of leukocytes, erythrocytes, and platelets levels of broilers according to the lighting regimen. The heterophil to lymphocyte ratio (H/L ratio) was 0.62, 0.51, 0.55, and 0.59 in each treatment; and was significantly lower in the 18L:6D treatment than that in the 24L treatment (*p* < 0.05). However, no significant differences were observed among the 24L, 8L:16D and 4L:2D treatments. Additionally, no significant differences were observed among the 18L:6D, 8L:16D and 4L:2D treatments.

Blood cells (leukocytes, erythrocytes, and platelets) are known as important indicators of broiler health status, including stress [21]. In particular, white blood cells protect against external stimuli and foreign substances in all vertebrates [22]. The H/L ratio is typically used as an indicator of stress in chickens [23].

It is known that continuous light, such as 24L or 23L:1D [4], or intermittent light [24] regimens disrupt resting behavior and increases stress in chickens. In a study by Olanrewaju et al. [25], the H/L ratio of birds under 8L:16D treatment was similar to that under 23L:1D and 2L:2D treatments; similar results were obtained in this study. The increased H/L ratio of birds under shorter light regimens (8L:16D) could be attributed to the stress experienced because of the limited accessibility to feed during the dark period. Lien et al. [1] reported that there was no significant difference in the H/L ratio between the 23L:1D and 18L:6D treatments. However, this could be because of the light intensity used by Lien et al. [1], which was 0.1 and 10 lx, whereas it was 35 lx in the present study.

### 3.3. Serum Biochemical Composition

Table 4 shows the results of the serum biochemical composition according to the lighting regimen involved. The total cholesterol (TC) in the 8L:16D treatment was 110.2 mg/dL, which was the lowest among the treatments (*p* < 0.05). The aspartate aminotransferase (AST) level in both 18L:6D and 8L:16D treatments was 277.2 U/L, which was significantly lower than that in other treatments (*p* < 0.001).

In the event of stress in poultry, the biological metabolism is altered, resulting in a decrease or increase in serum glucose, cholesterol, and albumin levels [26]. Therefore, the blood biochemical composition is often used as an indicator for evaluating metabolic diseases in poultry [27]. Aspartate aminotransferase (AST) is a major indicator of liver (inflammation, infection, etc.) and muscle (trauma, seizure, etc.) damage; increased AST results in cell damage [28]. In this study, higher AST levels in the 24L treatment than those in the 18L:6D and 8L:16D treatments were thought to be because of cell damage caused by physiological stress, as indicated in previous studies [4,24]. In contrast, blood glucose, triglycerides, and lactate dehydrogenase levels, which are related to feed intake, did not show a significant difference among the treatments; these results are in agreement with the work of Fidan et al. [13] and Olanrewaju et al. [29].

### 3.4. Cytokines and Corticosterone

The cytokine (IL-6, TNF-α) and corticosterone levels in the serum of broilers under different lighting regimens are shown in Table 5. IL-6 and corticosterone levels were significantly higher in the 24L and 4L:2D treatments (*p* < 0.001); we did not observe a significant difference in the TNF-α levels among the treatments.

With a prolonged photoperiod, the opportunity for rest and sleep in the birds decreases, which results in an increase in physiological stress, and the subsequent induction of inflammation [30]. Cytokines are released as a consequence of acute inflammation [31,32]. The release of serum corticosterone is activated by anxiety and stress; therefore, it may also be used as an indicator for stress in poultry [33]. Qin et al. [34] reported that the serum cytokine concentrations, including IL-6 and TNF-α, in ducks with FPD were significantly higher than those in healthy ducks. In the present study, it was presumed that IL-6 and corticosterone levels of broilers in the 24L and 4L:2D treatments increased because of the occurrence of FPD and physiological stress.

Various studies have analyzed changes in corticosterone concentrations, with respect to the photoperiod regimen in broilers. Buckland et al. [35] reported that plasma corticoid levels were significantly lower after 13 h of lighting than those after 24 h of lighting. Abbas et al. [36] reported that the serum corticosterone content was higher in birds under non-intermittent restricted light (12L:12D) than that under intermittent light (2L:2D).

The results of the present study that indicate increased cytokine and corticosterone levels under environmental stress conditions are, thus, consistent with those indicated by Buckland et al. [35] and Abbas et al. [36]. This is in accordance with the results of the H/L ratio in the current study. Pandey [37] reported that broilers require at least 4 h of resting time. However, stress levels may increase because of lower feed intake when the dark period is prolonged.

### 3.5. Litter Moisture Content and Foot Pad Dermatitis

Table 6 shows the litter moisture content and FPD scores according to the lighting regimen. The litter moisture content did not differ significantly among the treatments. However, the FPD score was significantly lower in the 18L:6D and 8L:16D treatments than that in the 24L and 4L:2D treatments (*p* < 0.001).

FPD causes inflammation and necrosis of the plantar surface of the footpad in broilers. FPD causes pain and negatively affects poultry welfare [38]. Severe lesions may result in reductions in weight gain, owing to the pain-induced decreases in feed intake [39]. Thus, the FPD score is often used as an indicator of broiler welfare in European countries [40] and Korea [15].

The occurrence of FPD is positively correlated with the litter moisture content [41,42] and body weight of birds [43]. Meluzzi et al. [41] stated that photoperiod may influence litter moisture content. In addition, light intensity or photoperiod can affect the activity of birds, and consequently, the occurrence of FPD. Ferrante et al. [44] showed that a moderate photoperiod (16L:8D) resulted in the decreased occurrence of footpad lesions in broilers. Schwean-Lardner et al. [45] also reported that footpad lesion scores were higher under longer photoperiods. Karaarslan and Nazlıgül [46] reported that the incidence of FPD decreased in birds under 18 h of light, compared with that under 23 h of light. In contrast, Petek et al. [47] reported that litter moisture content and the incidence of FPD did not differ significantly difference between 24L and intermittent light (2L:2D). The results of these studies are consistent with the findings of the present study.

Additionally, the litter moisture content was higher in the 24L and 4L:2D treatments than that in the 18L:6D and 8L:16D treatments, although no significant difference was observed. This result is consistent with the body weight of the broilers in each treatment. As mentioned earlier, the photoperiod influences bird activity, which is a major factor that affects the occurrence of FPD. Continuous light or frequent alternations between light and darkness stimulates the physical activity of birds. Therefore, it was assumed that physical activity according to the photoperiod, body weight gain, and presumably litter moisture content affected the overall occurrence of FPD in our study. However, the relationships among these factors need to be investigated further in future studies.

### 3.6. Carcass Characteristics and Physicochemical Properties of Breast Meat

Table 7 shows the carcass yield and carcass cut yields of broilers according to the lighting regimen. The photoperiod did not significantly affect carcass yield or carcass cut yields of broilers. Previous studies have shown that the photoperiod has little effect on carcass yield [1,6,13] or carcass cut yields, with consistent wings, legs and breast meat proportions [6,13]. The results of the present study were consistent with those of previous studies.

Table 8 shows the proximate composition and physicochemical properties of the breast meat according to the lighting regimen. No significant differences were detected in the moisture (76.1% to 76.5%), crude protein (21.9% to 22.2%), and crude fat (1.25% to 1.43%) contents of breast meat among the treatments. These proximate compositions were similar to the results of Kim et al. [48], who compared the breast meat characteristics between conventional and animal welfare farms. Tuell et al. [49] reported that moisture, crude protein, and crude fat contents were not affected by the photoperiod.

We did not observe any significant differences in meat quality characteristics, such as pH, meat color (L*, a*, b*), cooking loss, and WHC, among the treatments. However, the SF was significantly higher in the 24L treatment than in the 8L:16D treatment (*p* < 0.05). Meat color is used as an important indicator for consumers during purchase [50]. WHC and cooking loss indicate the ability to retain moisture during the processing and storage of meat and affect juiciness [51]. SF is associated with connective tissue that contributes to the meat preference, flavor, and tenderness of cooked meat [52]. Various studies have shown that the photoperiod does not affect the pH, meat color, cooking loss, or WHC of breast meat in broilers [13,47,49].

Kim et al. [48] reported that the SF of breast meat is significantly higher for broilers reared on welfare farms than for those reared in conventional farms. Other studies have also found that the SF of meat increased in birds reared in free-range systems [53,54]. They attributed this to the increased activity or movement of the broilers. In addition, in this study, it was assumed that the SF was affected by the higher activity of birds in the 24L treatment than in the 8L:16D treatment.

## 4. Conclusions

The overall results suggest that the photoperiod of 18L:6D recommended by the welfare standard is appropriate, considering the performance parameters and stress of broilers. When the performance of broilers improved with the prolonged photoperiod, it resulted in increased stress, whereas when the stress levels decreased with the reduced photoperiod, the performance also decreased. The optimum photoperiod for broilers may vary with age with respect to performance and welfare. Since an ideal photoperiod that satisfies both productivity and welfare has not yet been established, further research is needed to evaluate the various light regimens for their implementation in the broiler industry.

## Figures and Tables

**Table 1:** Footpad dermatitis score indicative of welfare.

Score	Contents
0	No foot pad dermatitis
1	Condition in which black necrosis and swelling is inconspicuous
2	Condition in which black necrosis and swelling are conspicuous, and inflammation is less than 25%
3	Condition in which the sole of the foot is enlarged due to swelling, and black necrosis affects less than 50% of the sole
4	The same as that of score 3, but black necrosis exceeds 50%

**Table 2:** Effect of photoperiod on growth performance of broilers.

Parameter	Photoperiod (Light:Dark)				SEM ^1^	*p*-Values
	24L	18L:6D	8L:16D	4L:2D		
Initial weight (g/bird)	158.9	155.4	157.7	157.2	1.99	0.667
Final weight (g/bird)	2083 ^a^	1968 ^ab^	1862 ^b^	2067 ^a^	45.9	0.018
Weight gain (g/bird)	1924 ^a^	1812 ^ab^	1705 ^b^	1910 ^a^	45.9	0.019
Feed intake (g/bird)	3354 ^ab^	3315 ^ab^	3117 ^b^	3389 ^a^	62.9	0.042
FCR (feed/gain)	1.78	1.94	1.92	1.85	0.043	0.095

^1^ SEM, standard error of means (n = 84). ^a,b^ Means in same rows with different superscripts are significantly different (*p* < 0.05). FCR, feed conversion rate.

**Table 3:** Effect of photoperiod on leukocyte, erythrocyte, and platelet profiles of broilers.

Parameters		Photoperiod (Light:Dark)				SEM ^1^	*p*-Values
		24L	18L:6D	8L:16D	4L:2D		
Leukocytes	WBC, K/μL	21.6	19.6	20.0	21.2	0.873	0.309
	HE, K/μL	7.14	5.53	6.10	6.61	0.447	0.083
	LY, K/μL	11.4	10.8	10.9	11.1	0.371	0.640
	H/L ratio	0.62 ^a^	0.51 ^b^	0.55 ^ab^	0.59 ^ab^	0.026	0.022
	MO, K/μL	2.28	2.02	2.13	2.10	0.082	0.163
	EO, K/μL	0.96	0.76	0.85	0.87	0.079	0.366
	BA, K/μL	0.33	0.24	0.28	0.31	0.036	0.363
Erythrocyte	RBC, K/μL	2.19	2.15	2.18	2.09	0.051	0.535
	Hb, g/dL	8.06	7.71	7.87	7.84	0.181	0.595
	HCT, %	21.9	21.4	21.5	21.7	0.571	0.918
	MCHC, g/dL	36.7	36.3	35.6	36.1	0.407	0.358
Platelets		16.5	12.8	13.7	13.8	2.177	0.659

^1^ SEM, standard error of means (n = 12). ^a,b^ Means in same rows with different superscripts are significantly different (*p* < 0.05). WBC, white blood cells; HE, heterophils; LY, lymphocytes; H/L, heterophils to lymphocytes; monocytes, MO; EO, eosinophils; BA, basophils; RBC, red blood cells; Hb, hemoglobulin; HCT, hematocrit; MCV, mean corpuscular volume; MCH, mean corpuscular hemoglobulin; MCHC, mean corpuscular hemoglobulin concentration.

**Table 4:** Effect of photoperiod on serum biochemical composition of broilers.

Parameters	Photoperiod (Light:Dark)				SEM ^1^	*p*-Values
	24L	18L:6D	8L:16D	4L:2D		
TC (mg/dL)	120.4 ^ab^	118.7 ^ab^	110.2 ^b^	125.2 ^a^	3.289	0.020
TG (mg/dL)	41.9	42.7	40.5	45.1	1.883	0.372
GLU (mg/dL)	176.0	156.9	165.1	174.6	7.225	0.220
TP (g/dL)	2.83	2.87	2.99	3.01	0.064	0.128
AST (U/L)	327.1 ^a^	277.2 ^b^	277.2 ^b^	298.0 ^ab^	8.210	<0.001
ALT (U/L)	2.80	2.64	2.53	2.95	0.129	0.130
CREAT (mg/dL)	0.21	0.21	0.21	0.22	0.005	0.293
ALB (g/dL)	1.20	1.19	1.24	1.23	0.025	0.348
IP (mg/dL)	9.56	9.12	9.06	9.01	0.232	0.329
LD (U/L)	2975	2910	2856	2869	110.9	0.872

^1^ SEM, standard error of means (n = 12). ^a,b^ Means in same rows with different superscripts are significantly different (*p* < 0.05). TC, total cholesterol; TG, triglyceride; GLU, glucose; TP, total protein; AST, aspartate aminotransferase; ALT, alanine aminotransferase; CREAT, creatinine; ALB, albumin; IP, inorganic phosphorus; LD, lactate dehydrogenase.

**Table 5:** Effect of photoperiod on cytokines and corticosterone concentration of broilers.

Parameters	Photoperiod (Light:Dark)				SEM ^1^	*p*-Values
	24L	18L:6D	8L:16D	4L:2D		
Cytokines (pg/mL)						
IL-6	55.1 ^a^	42.1 ^b^	41.4 ^b^	54.9 ^a^	2.905	<0.001
TNF-α	71.6	70.3	68.2	71.3	2.054	0.643
Corticosterone (ng/mL)	16.5 ^a^	11.8 ^b^	12.6 ^b^	15.9 ^a^	0.784	<0.001

^1^ SEM, standard error of means (n = 12). ^a,b^ Means in same rows with different superscripts are significantly different (*p* < 0.05). IL-6, interleukin-6; TNF-α, tumor necrosis factor-α.

**Table 6:** Effect of photoperiod on litter moisture content and occurrence of footpad dermatitis (FPD) of broilers.

Parameters	Photoperiod (Light:Dark)				SEM ^1^	*p*-Values
	24L	18L:6D	8L:16D	4L:2D		
Litter moisture content (%)	41.9	34.4	37.1	42.5	3.346	0.304
FPD (Score)	1.70 ^a^	0.83 ^b^	0.63 ^b^	1.88 ^a^	0.125	<0.001

^1^ SEM, standard error of means (litter moisture, n = 4; FPD, n = 40). ^a,b^ Means in same rows with different superscripts are significantly different (*p* < 0.05).

**Table 7:** Effect of photoperiod on carcass yields of broilers.

Parameter (% of Live Weight)	Photoperiod (Light:Dark)				SEM ^1^	*p*-Values
	24L	18L:6D	8L:16D	4L:2D		
Carcass	73.8	73.4	73.9	74.3	0.66	0.786
Breast	22.8	23.3	23.3	24.1	1.65	0.274
Legs	22.0	21.6	22.2	22.6	0.39	0.349
Wings	7.04	7.35	7.43	6.80	0.182	0.072
Neck	3.78	4.04	4.15	4.45	0.192	0.121
Back	17.9	17.3	17.2	16.8	0.48	0.450

^1^ SEM, standard error of means (n = 12).

**Table 8:** Effect of photoperiod on physicochemical characteristics of breast meat of broilers.

Parameter		Photoperiod (Light:Dark)				SEM ^1^	*p*-Values
		24L	18L:6D	8L:16D	4L:2D		
Proximate composition (%)	Moisture	76.5	76.3	76.4	76.1	0.195	0.512
	Crude protein	22.2	22.2	22.0	21.9	0.199	0.735
	Crude fat	1.43	1.34	1.25	1.28	0.115	0.686
pH		5.78	5.77	5.71	5.75	0.023	0.193
Color	L*	56.9	57.9	57.6	57.3	0.865	0.866
	a*	1.70	1.35	1.54	1.79	0.167	0.298
	b*	6.98	7.05	7.50	7.48	0.408	0.716
Cooking loss (%)		20.4	19.5	20.0	20.4	0.865	0.879
WHC (%)		55.9	55.5	55.9	55.2	0.529	0.739
Shear force (N)		21.6 ^a^	17.6 ^ab^	15.4 ^b^	19.9 ^ab^	1.502	<0.05

^1^ SEM, standard error of means. ^a,b^ Means in same rows with different superscripts are significantly different (*p* < 0.05). WHC, water holding capacity.

## Data Availability

The datasets generated during and/or analyzed during the current study are available from the corresponding author upon reasonable request.
